# Cell-free DNA Predicts Prolonged Response to Multi-agent Chemotherapy in Pancreatic Ductal Adenocarcinoma

**DOI:** 10.1158/2767-9764.CRC-22-0343

**Published:** 2022-11-11

**Authors:** Eric S. Christenson, Su Jin Lim, Jennifer Durham, Ana De Jesus-Acosta, Katherine Bever, Daniel Laheru, Amy Ryan, Parul Agarwal, Robert B. Scharpf, Dung T. Le, Hao Wang

**Affiliations:** Bloomberg-Kimmel Institute, Sidney Kimmel Comprehensive Cancer Center, and The Skip Viragh Center for Pancreatic Cancer Research and Clinical Care, Johns Hopkins University School of Medicine, Baltimore, Maryland.

## Abstract

The treatment of metastatic pancreatic ductal adenocarcinoma (PDAC) is frequently characterized by significant toxicity and rapid development of resistance to current approved therapies. More reliable biomarkers of response are needed to guide clinical decision making.

We evaluated cell-free DNA (cfDNA) using a tumor-agnostic platform and traditional biomarkers (CEA and CA19-9) levels in 12 patients treated at Johns Hopkins University on NCT02324543 “Study of Gemcitabine/Nab-Paclitaxel/Xeloda (GAX) in Combination With Cisplatin and Irinotecan in Subjects With Metastatic Pancreatic Cancer.” The pretreatment values, levels after 2 months of treatment, and change in biomarker levels with treatment were compared with clinical outcomes to determine their predictive value.

The variant allele frequency (VAF) of *KRAS* and *TP53* mutations in cfDNA after 2 months of treatment was predictive of progression-free survival (PFS) and overall survival (OS). In particular, patients with a lower-than-average *KRAS* VAF after 2 months of treatment had a substantially longer PFS than patients with higher posttreatment *KRAS* VAF (20.96 vs. 4.39 months). Changes in CEA and CA19-9 after 2 months of treatment were also good predictors of PFS.

Comparison via concordance index demonstrated *KRAS* or *TP53* VAF after 2 months of treatment to be better predictors of PFS and OS than CA19-9 or CEA. This pilot study requires validation but suggests cfDNA measurement is a useful adjunct to traditional protein biomarkers and imaging evaluation and could distinguish between patients who are likely to achieve prolonged responses versus those that will have early progression and may benefit from a change in treatment approach.

**Significance:**

We report on the association of cfDNA with response durability for patients undergoing treatment with a novel metronomic chemotherapy regimen (gemcitabine, nab-paclitaxel, capecitabine, cisplatin, irinotecan; GAX-CI) for metastatic PDAC. This investigation offers encouraging evidence that cfDNA may prove to be a valuable diagnostic tool to guide clinical management.

## Introduction

Pancreatic cancer represents the fourth leading cause of cancer death in the United States with an estimated 48,220 pancreatic cancer–related deaths in 2021 which is driven in part by the highest case-fatality rate of any solid tumor malignancy (1). The mainstay of current treatment for metastatic pancreatic adenocarcinoma (PDAC) in patients with a good performance status is combination chemotherapy with either gemcitabine and nab-paclitaxel or 5-flurouracil, irinotecan, and oxaliplatin (2–4). While these combination chemotherapy regimens substantially improve progression-free survival (PFS) and overall survival (OS) compared to single-agent gemcitabine, they still result in a median OS of less than 12 months, highlighting the urgent need for more effective treatment strategies (2, 3).

When one of these two combination regimens is no longer effective in controlling a patient's cancer, the current treatment paradigm involves transitioning the patient to whichever of these regimens was not yet utilized (5–8). However, this strategy is frequently prevented by the rapid decline of patient functional status accompanying disease progression rendering the patient unable to tolerate more than one line of aggressive combination chemotherapy. This dilemma raises enthusiasm for (i) better targeted and/or more intensive treatment regimens to more effectively prevent disease progression and (ii) earlier identification of therapeutic resistance to allow more timely transition to alternative treatment approaches.

We hypothesized that combining the drug classes from the two most active first-line approaches into a five drug combination (gemcitabine, nab-paclitaxel, capecitabine, cisplatin, and irinotecan abbreviated GAX-CI) would maximize therapeutic responses and delay the onset of therapeutic resistance and performance status deterioration. To investigate this combination, we launched a phase Ib/II trial in which 47 patients were treated with this approach including 30 patients at the stage II expansion cohort dosing. We used this trial as a platform to also explore the use of biomarker strategies to distinguish which patients are likely to achieve favorable clinical outcomes.

Traditionally, protein-based biomarkers (CEA and CA19-9) have served as adjuncts to CT imaging for assessing the response of patients to treatment, an approach that has yielded limited incremental benefit in predicting patient outcomes. More recently, cell-free DNA (cfDNA) has emerged as a valuable tool to characterize and quantify tumor-specific genetic alterations shed into the bloodstream by cancer cells (9, 10). This tool has shown promise for the early identification of treatment response, recognition of disease progression, and describing potential mechanisms of treatment resistance. To assess the predictive ability of cfDNA and compare with CEA and CA19-9, we analyzed biomarker values in a subgroup of 12 patients in samples obtained pretreatment and after 2 months of treatment to determine how well these factors predict clinical outcomes.

## Materials and Methods

### Study Cohort

This study explored the utility of cfDNA to predict clinical treatment outcomes in patients with metastatic PDAC. The patients in this investigation were treated at Johns Hopkins University (JHU, Baltimore, MD) on NCT02324543 “Study of Gemcitabine/Nab-Paclitaxel/Xeloda (GAX) in Combination With Cisplatin and Irinotecan in Subjects With Metastatic Pancreatic Cancer” with five different drugs active against PDAC in an effort to maximize depth and duration of response. Patients with untreated metastatic PDAC with Eastern Cooperative Oncology Group performance 0 or 1 were eligible for this trial and were excluded if they were considered candidates for surgery, or had received prior chemotherapy or radiation previously. The study was conducted in accordance with the guidelines of the Declaration of Helsinki and approved by the JHU Institutional Review Board. All patients provided written informed consent prior to enrollment. From this cohort, we chose 12 patients (8 female, 4 male) to perform an investigation into the ability of biomarkers to predict treatment outcomes. Demographic information is available in Supplementary Table S1. Six patients with prolonged PFS (median: 14.1 months, range: 7.4–29.7 months) and 6 patients with a shorter PFS (median: 3.7 months, range: 2–5.6 months) were included. The selection of patients from the extremes of clinical outcomes improved statistical power to detect differences and was done without knowledge of biomarker levels.

### Biomarker Assessment

We obtained blood draws pretreatment and after 2 months of treatment (two cycles of GAX-CI) at the time of treatment evaluation with CT imaging. CEA and CA19-9 levels were assessed by the Johns Hopkins Pathology Department using standard laboratory practices and a Clinical Laboratory Improvement Amendments (CLIA)-approved Tosoh Bioscience Immunoassay method. cfDNA was obtained in cfDNA BCT blood collection tubes (Streck). cfDNA was stored at room temperature and isolated within 48 hours of collection using the Qiagen QIAam MinElute cfDNA Midi Kit (Qiagen). Following isolation, cfDNA was stored at −80°C prior to transfer to Tempus for use on the Tempus xF 105 gene liquid biopsy platform (Tempus). This assay is a hybrid-capture, next-generation sequencing assay that evaluates single-nucleotide variants and insertions and/or deletions at a minimum of 1,000× coverage (11). Reads were compared with a consensus genome and deviations cataloged along with their frequency relative to total number of reads at that gene locus to calculate variant allele frequency (VAF). Tissue-based molecular sequencing obtained as part of standard of care on CLIA-approved platforms was available for 9 of the 12 patients assessed. These were recorded and compared with cfDNA results.

### Determine Correlation between CEA, CA19-9, cfDNA Biomarkers, and CT Response

VAF of pathogenic *KRAS* and *TP53* alleles as well as the overall median VAF of all detected mutations in the cfDNA samples were used for analysis (12, 13). Values before treatment (pretreatment) and after two cycles of treatment (posttreatment), and the change in VAF between pretreatment and posttreatment measurements, as a percent change were evaluated. Percent change was defined as [(post − pre)/pre] × 100 as a measure of relative change divided by the pre-value × 100. As the VAF of mutation alleles commonly decreased following two cycles of GAX-CI, this percent change was often negative. These measurements were compared with traditional protein-based biomarkers (CEA, CA19-9) for the same timepoints.

Each of the respective biomarkers was correlated with best radiographic response based on CT scan as defined by best percent change in target lesions without progressive disease in nontarget lesions. CT scans were obtained at baseline and at 2-month intervals throughout the course of treatment with measurement of response by change in target lesion diameter. Spearman rank correlation coefficient was calculated to determine the correlation between biomarker timepoints (pretreatment, posttreatment, percent change in biomarker levels) and CT response.

### Correlating Biomarkers with Survival Outcomes

Each of the respective biomarkers was correlated with clinical outcomes including PFS and OS. We performed Cox regression analysis to assess relationships between *KRAS*, *TP53*, and median tumor VAFs as continuous variables and survival outcomes. In addition, comparisons were made between patients with biomarker levels in the upper and lower half of those measured for each timepoint using log-rank tests. Kaplan–Meier curves were created for each biomarker's pretreatment, posttreatment, and change in biomarker levels for PFS and OS, respectively. Comparisons of predictive ability of cfDNA strategies and CEA or CA19-9 were made using the concordance index (c-index) for the same timepoints, or the percent changes pretreatment to posttreatment. Statistical tests were two sided, and *P* values equal to or less than 0.05 were considered statistically significant. Statistical analyses were performed using R version 4.1.0.

### Data Availability

The data generated in this study are not publicly available due to patient privacy considerations but are available upon reasonable request from the corresponding author.

## Results

For all 12 patients assessed, tumor-associated mutations were detected in either their pretreatment or posttreatment cfDNA samples. *KRAS* mutations were detectable in the cfDNA of 11 of the 12 patients pretreatment and 7 of the 12 patients posttreatment, including the 1 patient without pretreatment positivity. *TP53* mutations were detectable in the cfDNA of 8 of the 12 patients pretreatment, and 7 of the 12 posttreatment, including one that was negative pretreatment (Supplementary Table S2). Of the 3 patients with no detectable *TP53* mutations in their plasma at either timepoint, only 1 harbored a *TP53* mutation in their tumor. This patient was assigned a *TP53* VAF value of 0 for both timepoints as well as percent change. The other 2 patients were excluded from *TP53* specific analyses.

### Relationship between Traditional Biomarkers and cfDNA

To determine the relationship between CEA, CA19-9, and cfDNA, the degree of correlation was measured using Spearman rank correlation coefficient. This was performed using pretreatment and posttreatment values and the percent change between these pretreatment and posttreatment measurements. A Spearman rank correlation coefficient of CEA and *KRAS* VAF was weak at pretreatment, posttreatment as well as for percent change (*R* = 0.36, 0.35, 0.28, respectively). There was a moderate correlation between CA19-9 and *KRAS* VAF at pretreatment and posttreatment and for the percent change (*R* = −0.35, 0.54, 0.48, respectively). A similar pattern was observed for the correlation between CEA and *TP53* VAF and between CA19-9 and *TP53* at either timepoint and for the percent change between timepoints (Supplementary Table S3). Pairwise scatterplots were created to visually represent correlations (Supplementary Fig. S1)

### Relationship between CT Response and Biomarkers

To determine the ability of KRAS and TP53 VAF to predict total tumor burden, the baseline tumor volume as measured by total RECIST measurements of target lesion diameters (in mm) was compared to baseline KRAS and TP53 VAFs. This analysis revealed no significant correlation between either KRAS or TP53 VAF and baseline tumor burden (Supplementary Fig. S2). The percent change in target lesion was correlated with change in each biomarker using Spearman rank correlation coefficient. This analysis revealed a statistically significant correlation between CT response and *KRAS* VAF at posttreatment (KRAS2) and change in *KRAS* VAF (KRASΔ; Table 1; Supplementary Fig. S3). There was also no statistically significant relationship between *TP53* VAF, CEA, or CA19-9 measurements at either timepoint and for percent change in biomarkers and CT responses (Table 1).

**TABLE 1:** Spearman rank correlation coefficient (*R*) between CT responses and biomarkers (CEA, CA19-9, *TP53* VAF, and *KRAS* VAF) at pretreatment, posttreatment (after two cycles of GAX-CI) and percent change between these two timepoints

	CEA		CA19-9		*KRAS* VAF		*TP53* VAF	
	*R*	*P*	*R*	*P*	*R*	*P*	*R*	*P*
**PreTx**	−0.14	0.664	−0.25	0.43	−0.38	0.224	−0.28	0.434
**PostTx**	0.36	0.256	0.07	0.834	0.58	0.048	0.58	0.077
**% change**	0.34	0.287	0.12	0.716	0.59	0.041	0.62	0.056

### Correlation between Biomarkers (CEA, CA19-9, and cfDNA) and Survival Outcomes

To determine the association between biomarker measurements and duration of benefit to GAX-CI as measured by PFS and OS, patients were stratified in half between those with a biomarker level or VAF above or at median (high) versus those with below median for each timepoint. For percent change between two timepoints, patients were stratified by whether they had a percent change above or at median (high) versus below median (low). As the majority of patients experienced decreases in mutation VAFs after two cycles, the low group values were more negative denoting larger drops in mutation fractions. Survival outcomes were compared between two groups using log-rank test.

**FIGURE 1:**
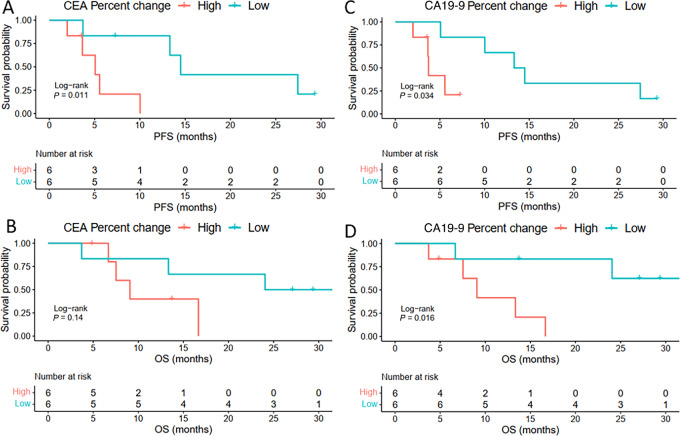
Change in CA19-9 levels but not CEA correlation with PFS and OS. The change in these levels was the percent change from baseline to after two cycles of GAX-CI, and patients were stratified by whether they had a change at or above median (high) or below median (low). This demonstrated a statistically improved PFS for patients with CEA (**A**) change below median (low CEA) but not for OS, (**B**) and CA19-9 showed statistically significant improvements in PFS (**C,** low 13.90 months vs. high 3.71 months) and OS (**D,** low not reached vs. high 9.07 months) with CA19-9 change below median (low CA19-9).

While pretreatment and posttreatment levels of CEA and CA19-9 did not predict PFS and OS, the change in CEA with treatment significantly correlated with PFS but not OS while change in CA19-9 was predictive of both (Fig. 1).

**FIGURE 2:**
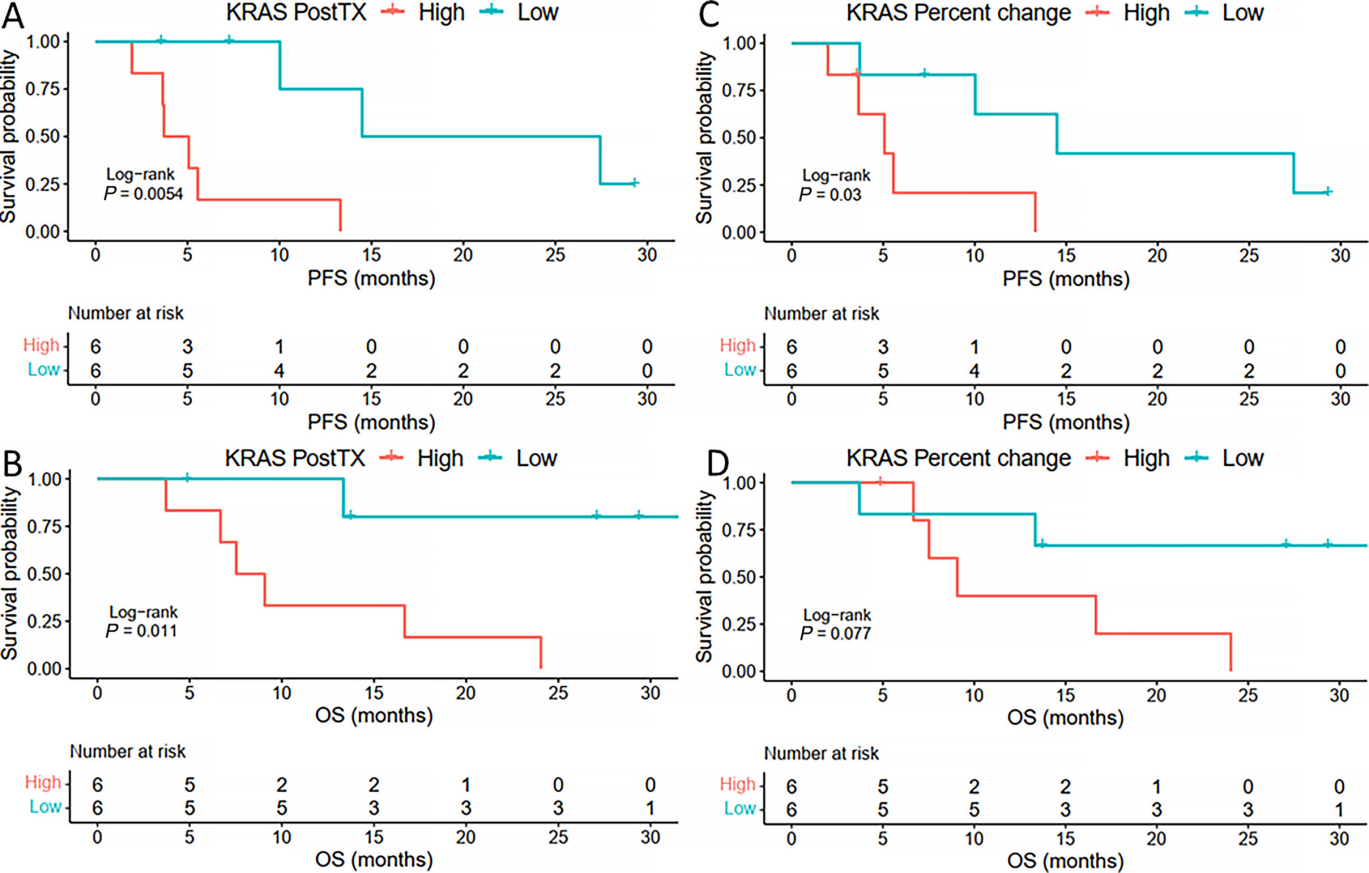
Posttreatment *KRAS* VAF and change in VAF with treatment predict survival outcomes. The change in *KRAS* VAF was the percent change from baseline to after two cycles of GAX-CI. Patients were stratified by whether they had a higher (PostTX high) or lower (PostTX low) than median posttreatment *KRAS* VAF, and a larger (change high) or smaller (change low) than median of change in *KRAS* VAF with treatment. This demonstrated a statistically improved PFS (**A,** PostTX low: 20.96 months vs. PostTX high: 4.39 months) and OS (**B,** PostTX low: not reached vs. PostTX high: 8.3 months) for patients that had lower *KRAS* VAF after two cycles of GAX-CI. For change in *KRAS* VAF with two cycles of treatment, change in *KRAS* was only associated with a statistically improved PFS (**C,***KRAS* change high: 5.06 months vs. *KRAS* change low: 14.49 months) but not OS (**D**).

To assess whether cfDNA measurements were associated with PFS and OS, *KRAS* VAF was evaluated at different measurements including pretreatment, posttreatment with two cycles of GAX-CI, and change from pretreatment to posttreatment (Fig. 2). Pretreatment values between these two groups did not show an association with survival outcomes whereas KRAS2 and KRASΔ both correlated with significantly better PFS (Fig. 2). We looked *KRAS* VAF as a continuous variable and compared this with PFS and OS using Cox regression modeling (Table 2). Pretreatment *KRAS* VAF was associated with OS [HR: 1.07 (1.00–1.15), *P* = 0.036] while KRAS2 was associated with PFS [KRAS2 HR 1.52 (1.09–2.12), *P* = 0.013] (Table 2 for PFS and Supplementary Table S4 for OS).

**TABLE 2:** Comparing predictive value of *TP53* VAF, *KRAS* VAF, or median VAF versus CEA and CA19-9 for PFS using c-index

Markers	HR (univariable)	*P*	c-index
**Pretreatment**			
*TP53* VAF	0.99 (0.92–1.06)	0.757	0.5
*KRAS* VAF	1.02 (0.96–1.07)	0.546	0.559
Median VAF	1.00 (0.93–1.08)	0.971	0.549
CEA	1.00 (1.00–1.01)	0.407	0.392
CA19-9	1.00 (1.00–1.00)	0.548	0.431
**Posttreatment**			
*TP53* VAF	1.46 (1.06–2.02)	0.021	0.853
*KRAS* VAF	1.52 (1.09–2.12)	0.013	0.882
Median VAF	1.49 (1.08–2.07)	0.016	0.863
CEA	1.00 (1.00–1.01)	0.497	0.608
CA19-9	1.00 (1.00–1.00)	0.524	0.686
**% change**			
*TP53* VAF	1.01 (1.00–1.02)	0.246	0.735
*KRAS* VAF	1.01 (1.00–1.01)	0.187	0.765
Median VAF	1.01 (1.00–1.01)	0.266	0.676
CEA	1.01 (1.00–1.01)	0.044	0.725
CA19-9	1.00 (1.00–1.01)	0.032	0.804

**FIGURE 3:**
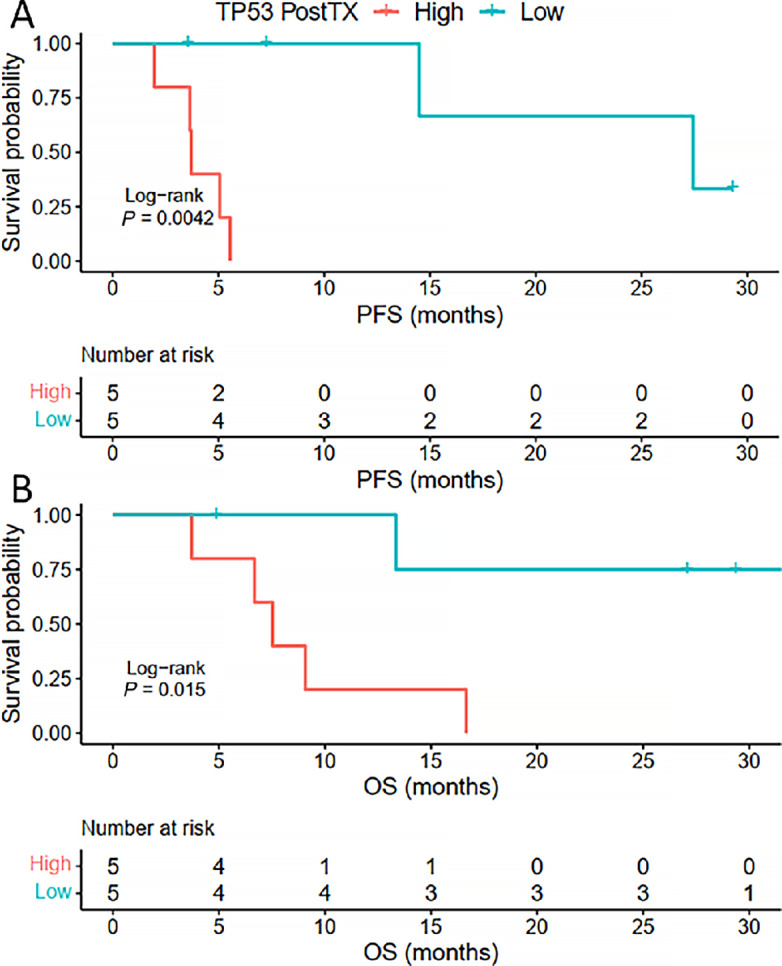
*TP53* VAF after two cycles of GAX-CI chemotherapy predicts PFS and OS. Patients were stratified by whether they had a higher (PostTX high) or lower (PostTX low) *TP53* VAF after two cycles of treatment. PFS (**A,** PostTX low: 27.43 months vs. PostTX high: 3.71 months) and OS (**B,** PostTX low: not reached vs. PostTX high: 7.52 months) was better for patients that had lower *TP53* VAF after two cycles of GAX-CI.

The association of *TP53* VAF in pretreatment and posttreatment cfDNA (*TP53*-2) with clinical outcomes was evaluated. Pretreatment values and change in VAF between these high and low groups did not show an association with OS or PFS whereas lower *TP53-*2 showed significantly improved OS and PFS (Fig. 3). We looked at *TP53* VAF as a continuous variable and compared this with PFS and OS using Cox regression modeling (Table 2). In this analysis, *TP53*-2 was associated with PFS [posttreatment HR: 1.46 (1.06–2.02), *P* = 0.021].

While *KRAS* and *TP53* mutations were both found in the majority of our evaluated patients, there were other mutations identified in individual patients including RB1, SMAD4, and STK11 pathogenic mutations (Supplementary Fig. S4). To incorporate this additional information into our analyses, we looked at the median tumor VAF across all identified mutations for each patient and/or timepoint as a marker of total tumor burden. We evaluated the median tumor VAF at pretreatment and posttreatment as well as change in median VAF between these two timepoints. While all of these analyses showed improved outcomes for patients with lower VAF, the only measurement that showed statistical significance was posttreatment median tumor VAF for PFS with lower median tumor VAF being associated with longer PFS (median PFS was 27.43 months in lower VAF and 5.06 months in higher group, *P* = 0.026; Supplementary Fig. S5).

### DNA Damage Repair Mutations

As DNA damage repair pathway mutations have been previously been associated with responses to platinum-based chemotherapy in PDAC, we assessed for the presence of these mutations in our patient population with identification of 3 patients with previously characterized pathogenic mutations in these pathways, BRCA2 V2969fs, BRCA2 Y1655*, and PMS2 E705K (14, 15). These patients had a longer PFS than the other patients (median OS: 28.8 vs. 5.6 months, *P* = 0.02 by log rank test), there was also a trend toward these patients having a longer OS.

### Comparing the Ability of Traditional Biomarkers and cfDNA to Predict PFS and OS

Univariate Cox regression analysis demonstrated a correlation between the change in CEA and CA19-9 during treatment and PFS in this cohort (Table 2). In contrast, levels of *TP53*, *KRAS*, and median tumor VAF after two cycles of treatment were correlated with PFS while the percent change in VAF during treatment was not predictive using this analysis. The predictive ability of biomarkers was compared against each other using c-index (Table 2). This demonstrated that while none of marker levels at pretreatment baseline predicted PFS well, *TP53*, *KRAS*, and median tumor VAF after two cycles of treatment were all strong predictors of PFS, and had a numerically higher c-index than CEA or CA19-9 at this timepoint. In contrast, while the early change in biomarker values of *KRAS*, *TP53*, or median tumor VAF after two cycles of treatment showed good prediction accuracy for PFS, the change of CA19-9 had a numerically higher c-index for predicting PFS. We also evaluated each biomarker's ability to predict OS outcome. *KRAS*2 and median tumor VAF after two cycles of treatment showed good predictive ability for OS (c-index = 0.79 and 0.70, respectively; Supplementary Table S3).

## Discussion

cfDNA is an emerging technology to characterize and quantify tumor-specific genetic alterations shed into the bloodstream by cancer cells (9, 10) . This approach has demonstrated promise for the detection of minimal residual disease, and to identify the presence of resistance mutations to targeted therapy (10, 16–22). Increasingly, there is an appreciation that the dynamic measurement of cfDNA levels can also provide an earlier measure of treatment response than currently utilized radiographic measures (23–26). This investigation demonstrated the potential utility of cfDNA to predict clinical outcomes in patients with metastatic PDAC treated with multi-agent chemotherapy. The clearance of *KRAS* or *TP53* following two cycles of GAX-CI was associated with a marked improvement in PFS and OS which could help inform the duration of treatment with this regimen. This finding is concordant with previously reported data in the neoadjuvant setting where absence of detectable cfDNA following PDAC treatment was associated with a substantially higher (80% vs. 38%) chance of achieving an R0 resection at the time of PDAC removal (27). While this investigation demonstrated agreement between trends in CEA, CA19-9, and cfDNA parameters, cfDNA offers several distinct advantages over more traditional biomarkers which argues for its use either as an adjunct to tumor antigen testing or to replace these protein measurements. Even in this relatively small treatment group, measurement of cfDNA mutation levels after two cycles of treatment showed stronger predictive value for PFS than CEA and CA19-9. In addition, approximately 6% of Caucasians and 22% of African Americans do not express CA19-9 on normal or tumor cells due to absence of the sialylated Lewis blood group antigen (28). Even when CA19-9 is expressed, its utility is hampered by significant variability of expression between patients for a given tumor volume thus requiring blood draws at multiple timepoints to have diagnostic value for therapeutic response.

The added value of cfDNA testing is likely to increase substantially in the near future. Mutation-directed therapy in PDAC remains in its infancy with only rarely identified genomic alterations (BRAF mutations, NTRK fusions) targetable by currently clinically FDA-approved agents (4, 29). However, recent success with KRAS G12C inhibitors highlights the expanding targeted therapy arsenal currently under development, including agents targeting *KRAS* and *TP53* mutations commonly seen in PDAC (30, 31). The use of targeted agents in clinical trials or as standard care will place increased importance on monitoring the clonal dynamics of tumors under the evolutionary pressures of treatment and examining the development of resistance through escape mutations.

Of note, the Tempus xF assay used for this investigation is a tumor-agnostic assay where a prespecified panel of genes was queried in the cfDNA specimen. This allowed for the identification of mutations even when these alterations are not detectable within the tissue specimen. A tumor-agnostic approach such as the one utilized in this investigation has several distinct advantages including not requiring a prior biopsy or resection specimen for completion which can be a limitation in sometimes challenging to biopsy pancreatic lesions. In contrast to a tumor-agnostic approach, tumor-informed approaches use tissue-based sequencing to identify molecular alterations in the tumor prior to development of a personalized cfDNA assay. This has a slower initial turnaround time as this assay has to be developed *de novo* but does potentially allow for improved sensitivity for detection of minimal residual disease by incorporating more of tumor-specific mutations specifically found in the patient's cancer while also helping to avoid “false positives” from clonal hematopoesis. Another potential advantage to a tumor-informed assay is it more readily facilitates the development of a multiple allele VAF approach that incorporates a panel of mutations to assess disease status. While this can also be considered with tumor-agnostic approach this was not investigated in our study given the small sample size evaluated.

This investigation has several limitations that must be considered when interpreting these results. First, this pilot study has only a limited number of patients with the intentional selection of 12 patients for evaluation which had disparate clinical outcomes (6 with short PFS and 6 with long PFS in the cohort). This increased our ability to demonstrate the utility of cfDNA in this disease context but means that these results particularly the magnitude of changes might not be representative to patients across the full range of clinical outcomes. Another limitation is 2 of our patients developed detectable *TP53*-2 that were not present preoperatively in either the cfDNA or tissue evaluation. This raises the possibility that these represented clonal hematopoiesis of indeterminate potential but given the uncertainty these were counted as true mutations with VAF recorded as such. These 2 patients had among the longest PFS so the approach we used may lead to an underestimation of the predictive value of *TP53* VAF. In addition, as this investigation was pursued in the context of a therapeutic trial, further studies are necessary to understand the associated predictive value in the standard-of-care treatment of PDAC or other treatment settings.

## Conclusion

In this investigation, we evaluated the ability of cfDNA to predict treatment outcomes in metastatic PDAC. This analysis identified that absolute cfDNA levels of the two most commonly identified mutations in our cohort (*KRAS* and *TP53*) after 2 months of treatment predicted PFS. This observation adds to the growing body of literature suggesting cfDNA might provide an early marker of treatment outcomes in patients with metastatic cancer.

## Supplementary Material

Figure S1 Supplemental Figure 1. Pairwise scatterplots comparing protein and cfDNA biomarkers for Post-treatment time point and % change pre-treatment to post-treatment. To determine the relationship between the different protein and cfDNA biomarkers we created pairwise scatterplots for the post-treatment time point (panel A) and % change (panel B). These data demonstrated good agreement between different cfDNA variables while the protein based showed variable correlations with cfDNA variables as outlined in Supplemental Table 3.

Figure S2 Supplemental Figure 2. Scatterplots comparing baseline RECIST tumor measurements with baseline cell-free DNA (cfDNA) biomarkers. To determine the relationship between baseline tumor mutational variant allele frequency (VAFs) and total tumor burden we compared baseline tumor burden as measured by RECIST measurements of evaluable lesions (sum of diameters in mm) with KRAS (panel A) and TP53 (panel B) VAFs for these tumors at the same time point. These data demonstrated no statistically significant relationship between baseline cfDNA VAF levels and RECIST measurements on CT scan.

Figure S3 Supplemental Figure 3. Post-treatment KRAS and change in KRAS VAF following treatment. To determine whether KRAS VAF following 2 cycles of treatment or the change in KRAS VAF from pre-treatment to after 2 cycles of treatment is correlated with CT response at 2 months, each patient’s CT response to treatment was plotted against KRAS VAF and calculated Spearman’s rank correlation coefficient. There was a significant correlation between post-treatment KRAS VAF and CT response (panel A) (R=0.58) and change in KRAS and CT response (panel B) (R=0.59)

Figure S4 Supplemental Figure 4. Spaghetti plot of tumor mutation VAF changes with 2 cycles of GAX-CI. To determine the impact of GAX-CI on the allelic frequency of mutations detected in ctDNA, we measured the VAF of these mutations pre-treatment and after 2 cycles of GAX-CI. The mutations detected for a given patient were plotted using a spaghetti plot to provide a visual representation of the change in VAF with treatment.

Figure S5 Supplemental Figure 5. Post-treatment Median VAF Predicts Progression-Free Survival. To assess whether Median mutation variant allele fraction (VAF) in cell-free DNA (cfDNA) predicted progression-free survival (PFS) and overall survival (OS), we sequenced cfDNA samples taken prior to treatment initiation and after 2 cycles of GAX-CI (on-treatment). The change in Median VAF was calculated was the percentage change from post- to pre-treatment. Patients were stratified by whether they had a higher (post-tx high) or lower (post-tx low) than the median post-treatment KRAS VAF, and a larger (change high) or smaller (change low) than the median of change in KRAS VAF with treatment. This demonstrated a statistically improved PFS with a lower post-treatment (after 2 cycles of treatment) Median mutation VAF (panel A). Post-treatment median mutation VAF did not predict OS (panel B). Change in Median mutation VAF with 2 cycles of treatment was not associated with a statistically improved PFS (panel C) or OS (panel D).

Tables S1-S4 Table S1: Patient demographics. Table S2: Summary of TP53 VAF and KRAS VAF data for patient cohort (N=12). Table S3: Correlation between TP53 VAF, KRAS VAF, or median VAF and CEA and CA19-9 biomarkers using Spearman rank correlation coefficient (R). Table S4: Comparing predictive value of TP53 VAF, KRAS VAF, or median VAF versus CEA and CA19-9 for OS using C-index
