# Supplementary figures and images for: Cell-free DNA Predicts Prolonged Response to Multi-agent Chemotherapy in Pancreatic Ductal Adenocarcinoma
Source: Cancer Res Commun. 2022 Nov 11;2(11):1418–25. doi: 10.1158/2767-9764.CRC-22-0343 (PMC10035498; doi:10.1158/2767-9764.CRC-22-0343)

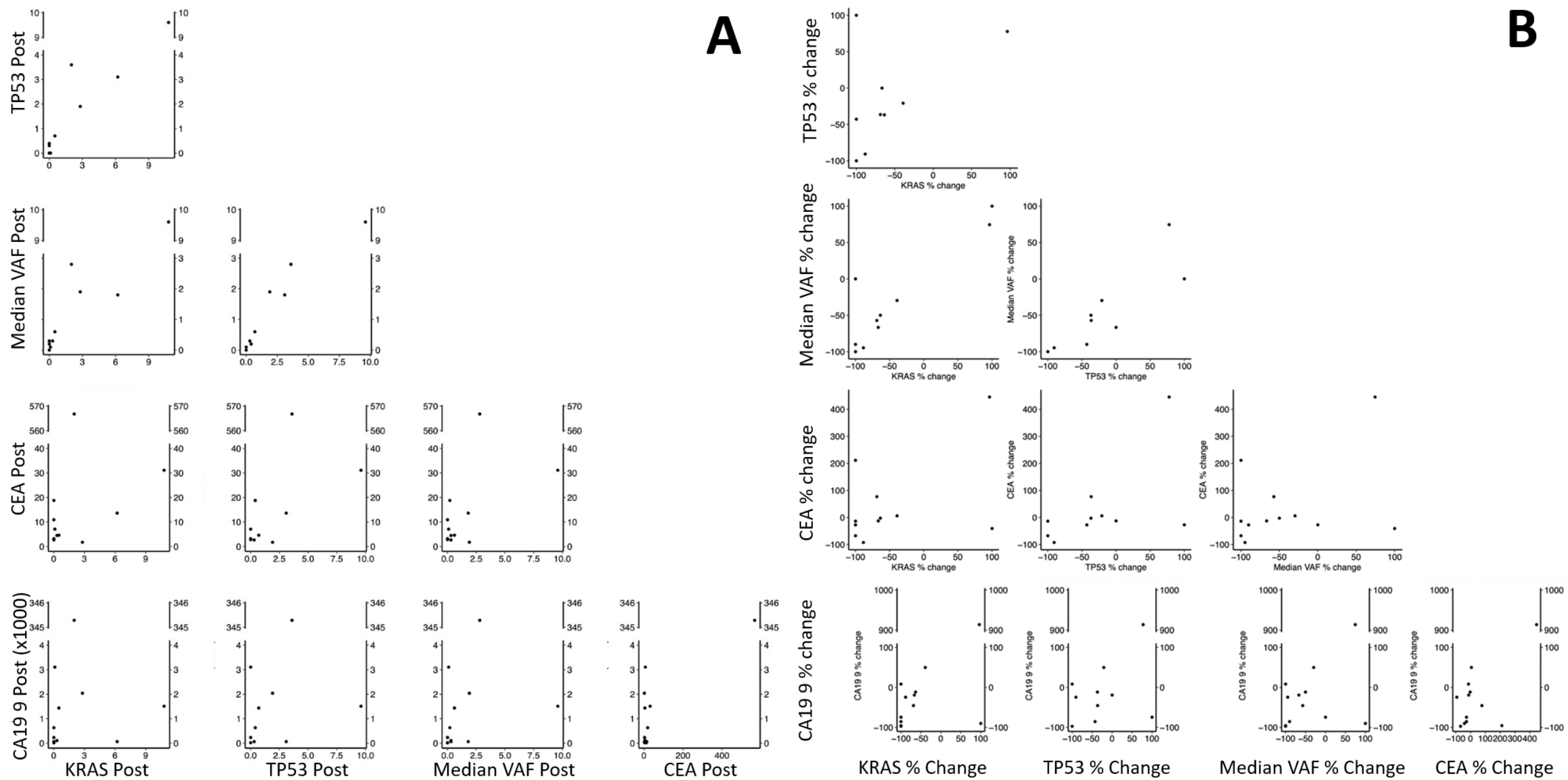

Supplemental Figure 1

Supplement: Figure S1 — Supplemental Figure 1. Pairwise scatterplots comparing protein and cfDNA biomarkers for Post-treatment time point and % change pre-treatment to post-treatment. To determine the relationship between the different protein and cfDNA biomarkers we created pairwise scatterplots for the post-treatment time point (panel A) and % change (panel B). These data demonstrated good agreement between different cfDNA variables while the protein based showed variable correlations with cfDNA variables as outlined in Supplemental Table 3. [file crc-22-0343-s01.pdf]

Supplemental Figure 2

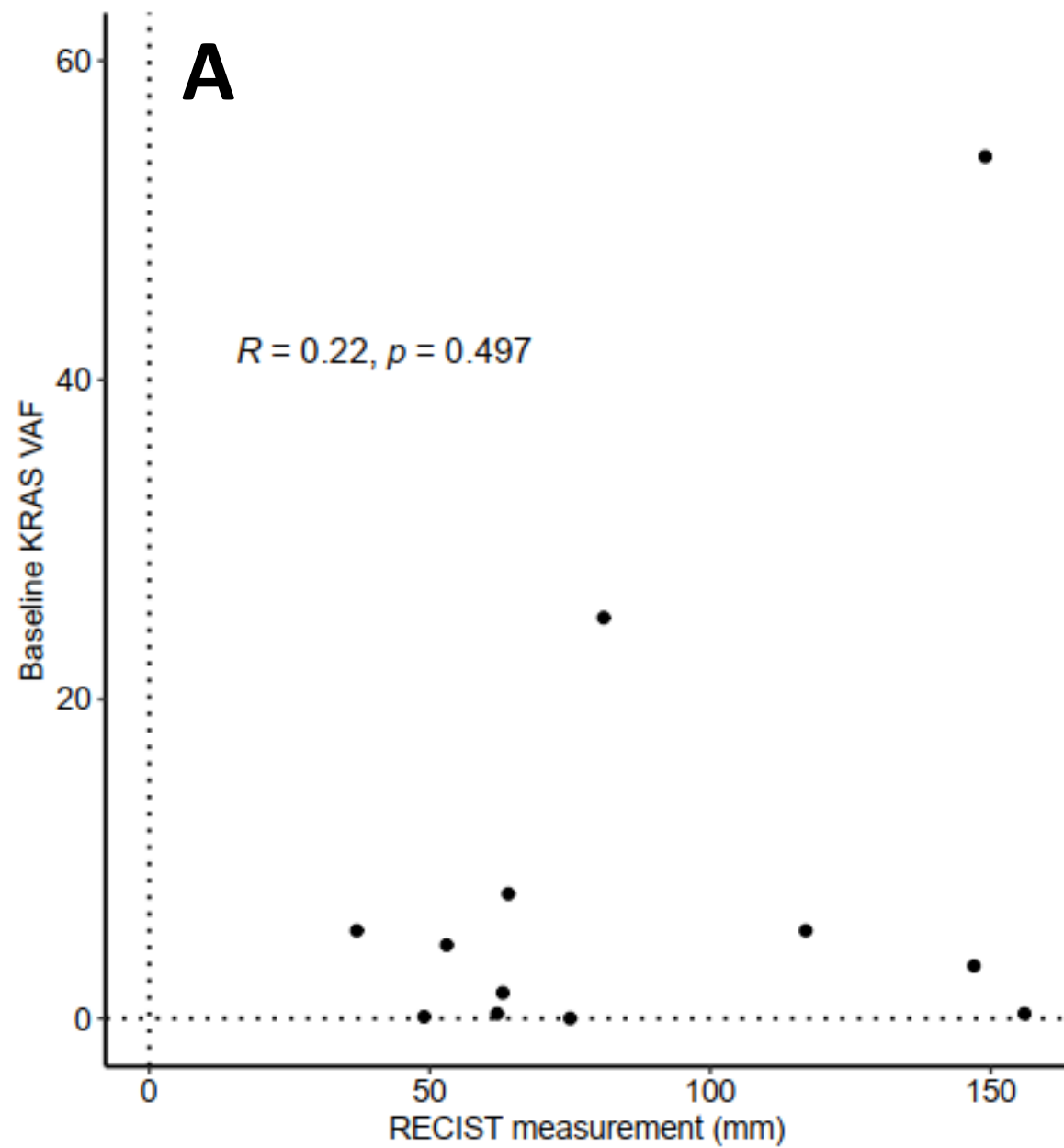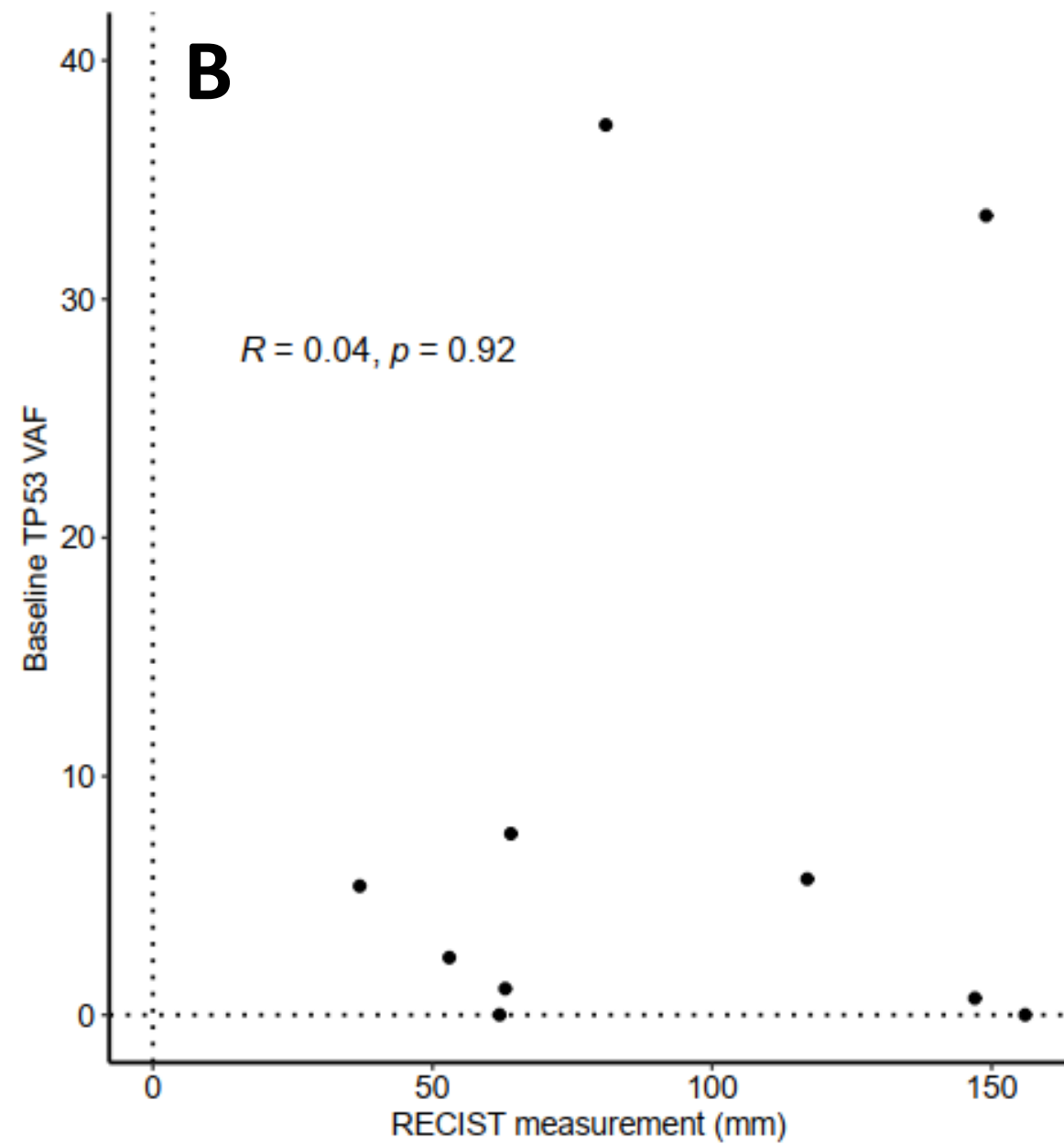

Supplement: Figure S2 — Supplemental Figure 2. Scatterplots comparing baseline RECIST tumor measurements with baseline cell-free DNA (cfDNA) biomarkers. To determine the relationship between baseline tumor mutational variant allele frequency (VAFs) and total tumor burden we compared baseline tumor burden as measured by RECIST measurements of evaluable lesions (sum of diameters in mm) with KRAS (panel A) and TP53 (panel B) VAFs for these tumors at the same time point. These data demonstrated no statistically significant relationship between baseline cfDNA VAF levels and RECIST measurements on CT scan. [file crc-22-0343-s02.pdf]

Supplemental Figure 3

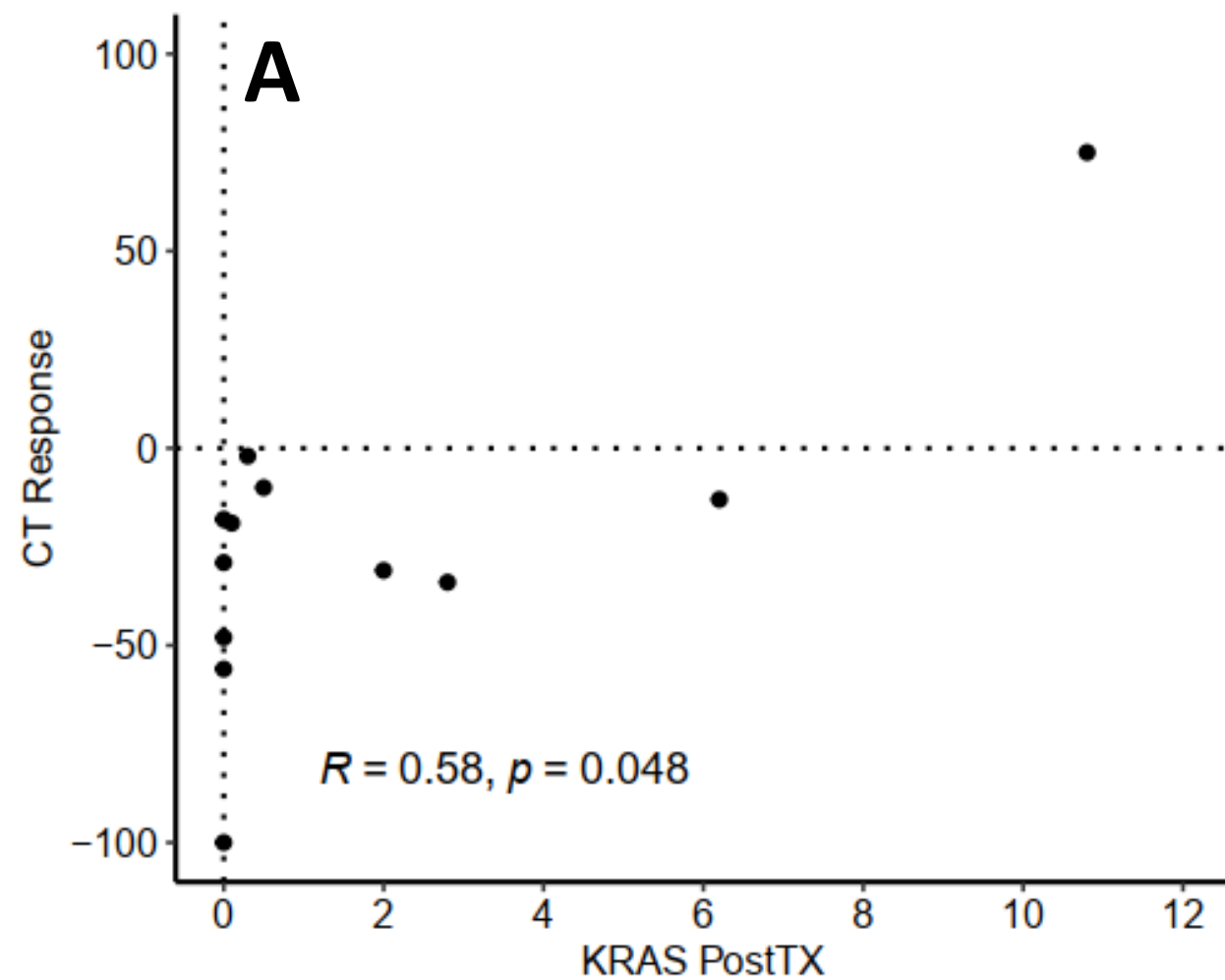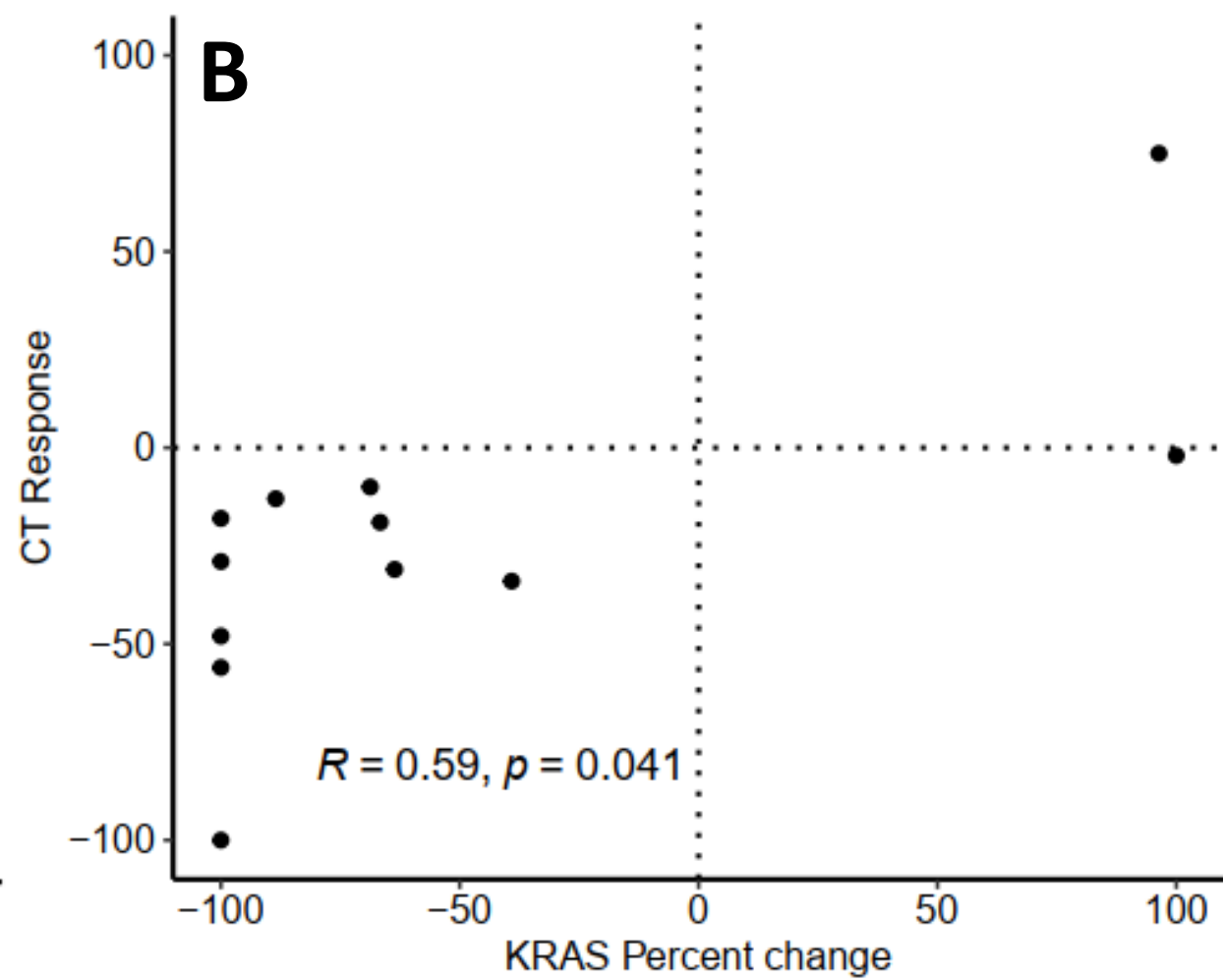

Supplement: Figure S3 — Supplemental Figure 3. Post-treatment KRAS and change in KRAS VAF following treatment. To determine whether KRAS VAF following 2 cycles of treatment or the change in KRAS VAF from pre-treatment to after 2 cycles of treatment is correlated with CT response at 2 months, each patient’s CT response to treatment was plotted against KRAS VAF and calculated Spearman’s rank correlation coefficient. There was a significant correlation between post-treatment KRAS VAF and CT response (panel A) (R=0.58) and change in KRAS and CT response (panel B) (R=0.59) [file crc-22-0343-s03.pdf]

Supplemental Figure 4

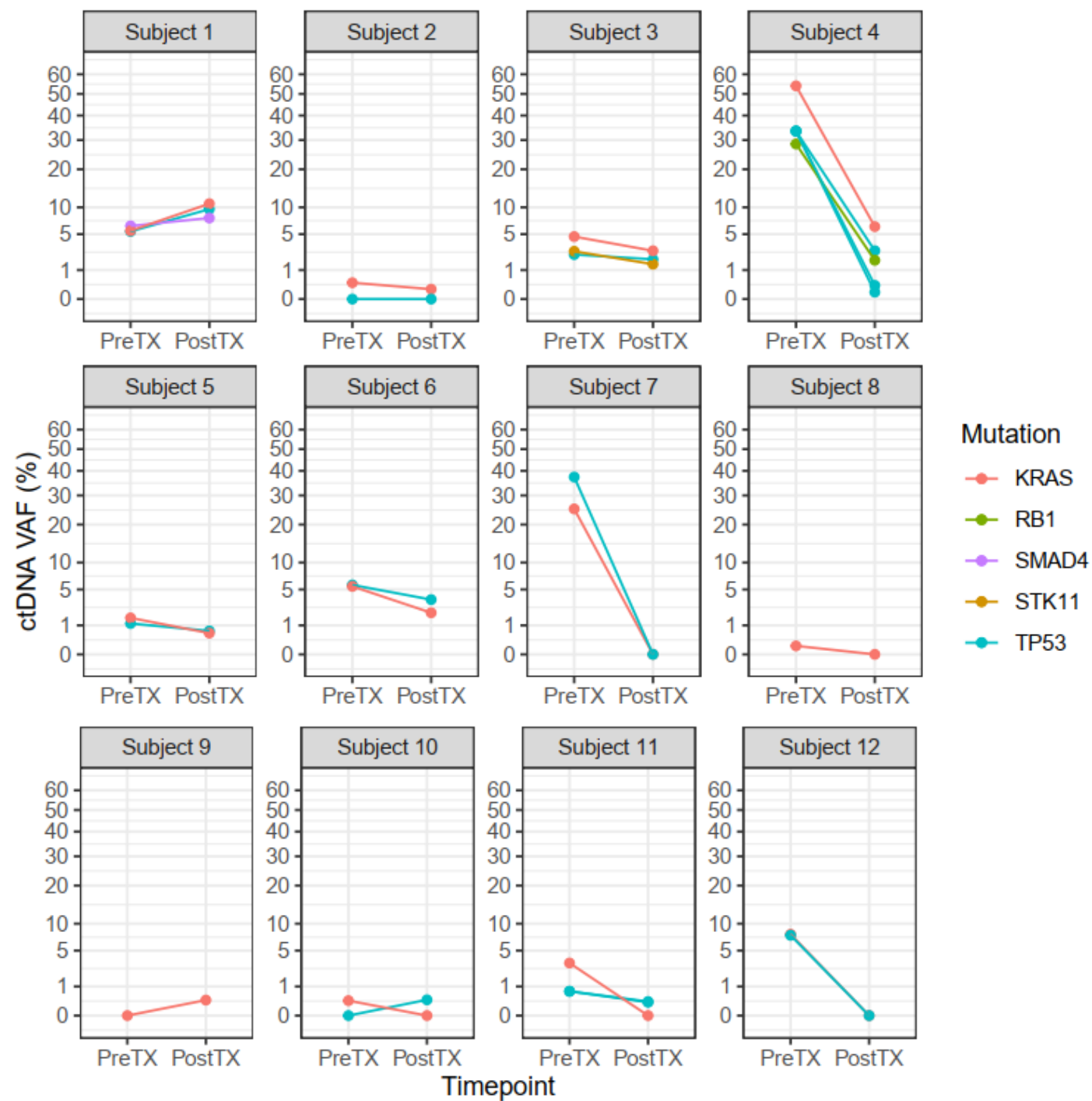

Supplement: Figure S4 — Supplemental Figure 4. Spaghetti plot of tumor mutation VAF changes with 2 cycles of GAX-CI. To determine the impact of GAX-CI on the allelic frequency of mutations detected in ctDNA, we measured the VAF of these mutations pre-treatment and after 2 cycles of GAX-CI. The mutations detected for a given patient were plotted using a spaghetti plot to provide a visual representation of the change in VAF with treatment. [file crc-22-0343-s04.pdf]

Supplemental  
Figure 5

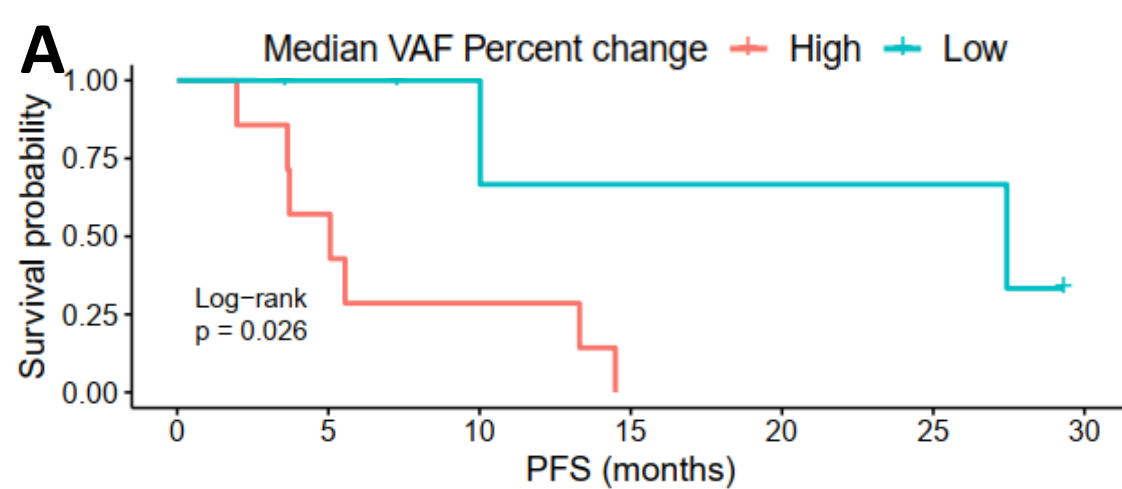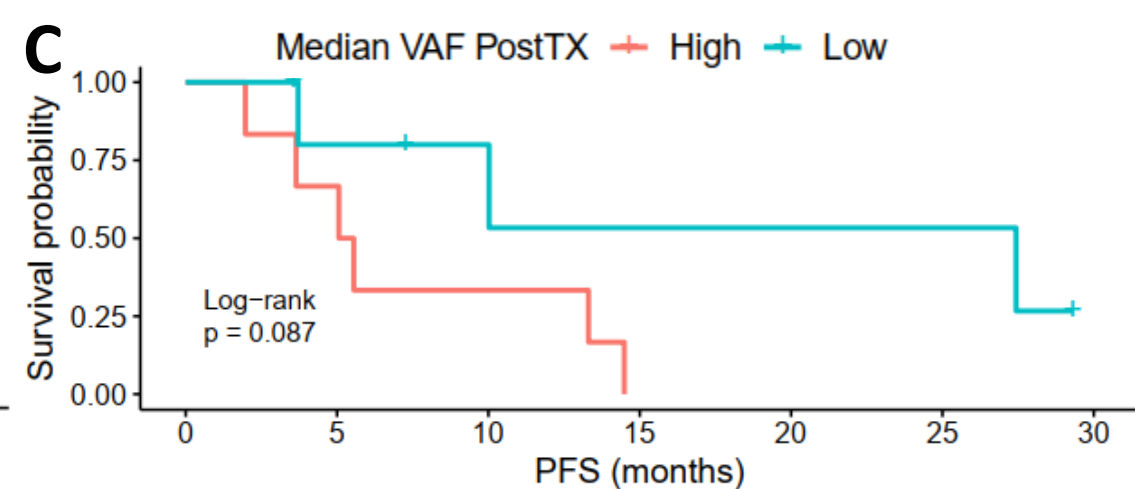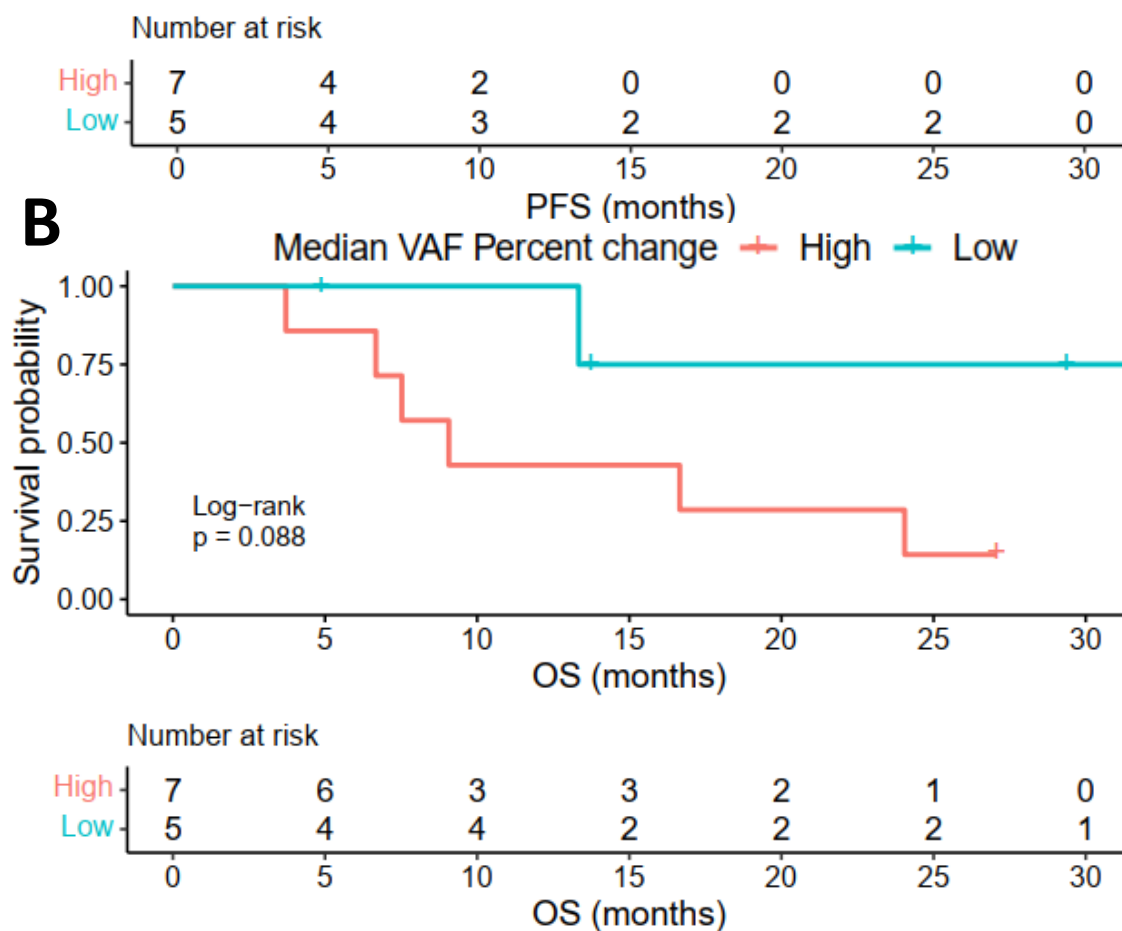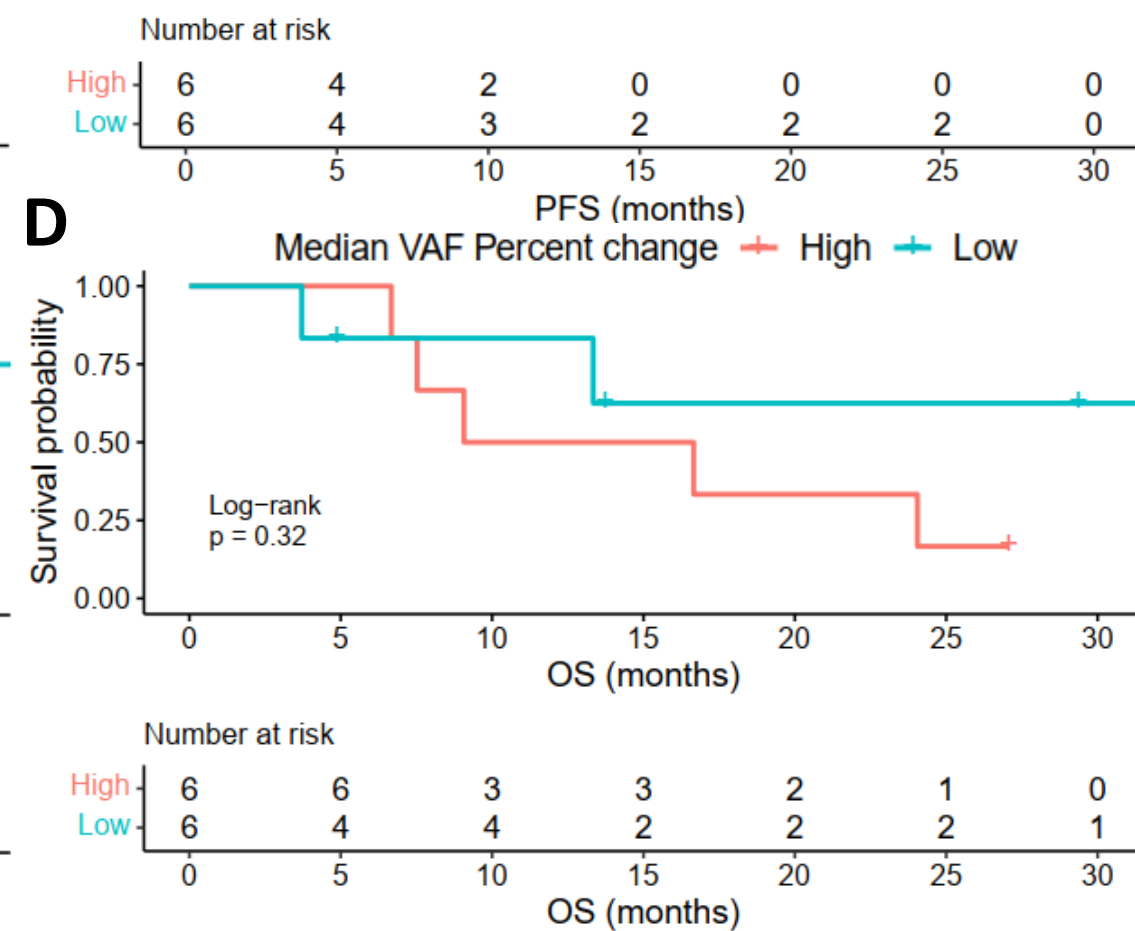

Supplement: Figure S5 — Supplemental Figure 5. Post-treatment Median VAF Predicts Progression-Free Survival. To assess whether Median mutation variant allele fraction (VAF) in cell-free DNA (cfDNA) predicted progression-free survival (PFS) and overall survival (OS), we sequenced cfDNA samples taken prior to treatment initiation and after 2 cycles of GAX-CI (on-treatment). The change in Median VAF was calculated was the percentage change from post- to pre-treatment. Patients were stratified by whether they had a higher (post-tx high) or lower (post-tx low) than the median post-treatment KRAS VAF, and a larger (change high) or smaller (change low) than the median of change in KRAS VAF with treatment. This demonstrated a statistically improved PFS with a lower post-treatment (after 2 cycles of treatment) Median mutation VAF (panel A). Post-treatment median mutation VAF did not predict OS (panel B). Change in Median mutation VAF with 2 cycles of treatment was not associated with a statistically improved PFS (panel C) or OS (panel D). [file crc-22-0343-s05.pdf]
