# Supplementary material for: Cell-free DNA Predicts Prolonged Response to Multi-agent Chemotherapy in Pancreatic Ductal Adenocarcinoma
Source: Cancer Res Commun. 2022 Nov 11;2(11):1418–25. doi: 10.1158/2767-9764.CRC-22-0343 (PMC10035498; doi:10.1158/2767-9764.CRC-22-0343)
Supplement: Tables S1-S4 — Table S1: Patient demographics. Table S2: Summary of TP53 VAF and KRAS VAF data for patient cohort (N=12). Table S3: Correlation between TP53 VAF, KRAS VAF, or median VAF and CEA and CA19-9 biomarkers using Spearman rank correlation coefficient (R). Table S4: Comparing predictive value of TP53 VAF, KRAS VAF, or median VAF versus CEA and CA19-9 for OS using C-index [file crc-22-0343-s06.docx]

Supplemental Table 1. Patient demographics.

|  | Total: n=12 | Long PFS (n=6) | Short PFS (n=6) |
| --- | --- | --- | --- |
| Age (years) |  |  |  |
| Mean (sd) | 58.2±9.2 | 59.5±9.0 | 56.8±9.3 |
| Median (sd) | 55±9.3 | 55.5±9.0 | 54±9.3 |
| Range | 47-74 | 51-74 | 47-70 |
| Gender (n, %) |  |  |  |
| Female | 8 (67) | 5 (83) | 3 (50) |
| Male | 4 (33) | 1 (17) | 3 (50) |
| Race (n, %) |  |  |  |
| African-American | 1 (8) | 1 (17) | 0 (0) |
| White | 11 (92) | 5 (83) | 6 (100) |
| Tumor Location (n, %) |  |  |  |
| Head | 8 (67) | 3 (50) | 5 (83) |
| Body/Tail | 4 (33) | 3 (50) | 1 (17) |
| Tumor Grade (n, %) |  |  |  |
| Moderately | 6 (50) | 4 (67) | 2 (33) |
| Moderate to poorly | 3 (25) | 2 (33) | 1 (17) |
| Poorly | 3 (25) | 0 (0) | 3 (50) |
| Sites of Metastasis (n, %) |  |  |  |
| Liver | 10 (83) | 4 (67) | 6 (100) |
| Lung | 3 (25) | 2 (33) | 1 (17) |
| Lymph nodes | 6 (50) | 4 (67) | 2 (33) |
| Other | 1 (8) | 1 (17) | 0 (0) |
| Peritoneum | 2 (17) | 1 (17) | 1 (17) |

Supplemental Table 2. Summary of *TP53* VAF and *KRAS* VAF data for patient cohort (N=12).

|  | **N=12** |
| --- | --- |
| ***TP53* PreTX** |  |
| Mean (SD) | 9.37 (14.0) |
| Median [Min, Max] | 3.90 [0, 37.3] |
| IQR [Q1, Q3] | 6.33 [0.800, 7.13] |
| Missing | 2 (16.7%) |
| ***TP53* PostTX** |  |
| Mean (SD) | 1.96 (2.99) |
| Median [Min, Max] | 0.550 [0, 9.60] |
| IQR [Q1, Q3] | 2.73 [0.0750, 2.80] |
| Missing | 2 (16.7%) |
| ***TP53* % change** |  |
| Mean (SD) | -25.0 (69.1) |
| Median [Min, Max] | -36.6 [-100, 100] |
| IQR [Q1, Q3] | 73.6 [-78.8, -5.21] |
| Missing | 2 (16.7%) |
| ***KRAS* PreTX** |  |
| Mean (SD) | 9.01 (15.7) |
| Median [Min, Max] | 3.95 [0, 54.0] |
| IQR [Q1, Q3] | 5.78 [0.300, 6.08] |
| ***KRAS* PostTX** |  |
| Mean (SD) | 1.89 (3.36) |
| Median [Min, Max] | 0.200 [0, 10.8] |
| IQR [Q1, Q3] | 2.20 [0, 2.20] |
| ***KRAS* % change** |  |
| Mean (SD) | -52.5 (73.1) |
| Median [Min, Max] | -78.6 [-100, 100] |
| IQR [Q1, Q3] | 42.5 [-100, -57.5] |

Supplemental Table 3. Correlation between *TP53* VAF, *KRAS* VAF, or median VAF and CEA and CA19-9 biomarkers using Spearman rank correlation coefficient (R).

| **ctDNA Marker** | **Protein Marker** | ***R*** | **p-value** |
| --- | --- | --- | --- |
| **Pre-treatment** |  |  |  |
| *TP53* | CEA | 0.25 | 0.481 |
| *TP53* | CA19-9 | -0.47 | 0.172 |
| *KRAS* | CEA | 0.36 | 0.25 |
| *KRAS* | CA19-9 | -0.35 | 0.263 |
| Median | CEA | 0.38 | 0.222 |
| Median | CA19-9 | -0.32 | 0.318 |
| **Post-treatment** |  |  |  |
| *TP53* | CEA | 0.55 | 0.102 |
| *TP53* | CA19-9 | 0.42 | 0.223 |
| *KRAS* | CEA | 0.35 | 0.268 |
| *KRAS* | CA19-9 | 0.54 | 0.068 |
| Median | CEA | 0.35 | 0.266 |
| Median | CA19-9 | 0.58 | 0.049 |
| **% change** |  |  |  |
| *TP53* | CEA | 0.56 | 0.093 |
| *TP53* | CA19-9 | 0.29 | 0.413 |
| *KRAS* | CEA | 0.28 | 0.373 |
| *KRAS* | CA19-9 | 0.48 | 0.116 |
| Median | CEA | 0.21 | 0.51 |
| Median | CA19-9 | 0.32 | 0.304 |

Supplemental Table 4. Comparing predictive value of *TP53* VAF, *KRAS* VAF, or median VAF versus CEA and CA19-9 for OS using C-index

| **Markers** | **HR (univariable)** | **p-value** | **C-index** |
| --- | --- | --- | --- |
| **Pre-treatment** |  |  |  |
| *TP53* VAF | 1.03 (0.98-1.09) | 0.218 | 0.618 |
| *KRAS* VAF | 1.07 (1.00-1.15) | 0.036 | 0.656 |
| Median VAF | 1.06 (0.99-1.12) | 0.088 | 0.667 |
| CEA | 1.00 (1.00-1.01) | 0.218 | 0.448 |
| CA19-9 | 1.00 (1.00-1.00) | 0.369 | 0.479 |
| **Post-treatment** |  |  |  |
| *TP53* VAF | 1.07 (0.88-1.31) | 0.507 | 0.662 |
| *KRAS* VAF | 1.12 (0.94-1.32) | 0.200 | 0.792 |
| Median VAF | 1.09 (0.89-1.34) | 0.381 | 0.698 |
| CEA | 1.00 (1.00-1.01) | 0.365 | 0.583 |
| CA19-9 | 1.00 (1.00-1.00) | 0.359 | 0.688 |
| **% change** |  |  |  |
| *TP53* VAF | 1.00 (0.98-1.01) | 0.514 | 0.544 |
| *KRAS* VAF | 1.00 (0.99-1.01) | 0.526 | 0.604 |
| Median VAF | 1.00 (0.99-1.01) | 0.829 | 0.479 |
| CEA | 1.00 (1.00-1.01) | 0.831 | 0.562 |
| CA19-9 | 1.00 (1.00-1.00) | 0.533 | 0.688 |
